# The topology of data: opportunities for cancer research

**DOI:** 10.1093/bioinformatics/btab553

**Published:** 2021-07-28

**Authors:** Ciara F Loughrey, Padraig Fitzpatrick, Nick Orr, Anna Jurek-Loughrey

**Affiliations:** School of Electronics, Electrical Engineering and Computer Science, Queen’s University Belfast, Belfast, BT9 5BN, UK; School of Electronics, Electrical Engineering and Computer Science, Queen’s University Belfast, Belfast, BT9 5BN, UK; Patrick G Johnston Centre for Cancer Research, Queen’s University Belfast, Belfast, BT9 7AE, UK; School of Electronics, Electrical Engineering and Computer Science, Queen’s University Belfast, Belfast, BT9 5BN, UK

## Abstract

**Motivation:**

Topological methods have recently emerged as a reliable and interpretable framework for extracting information from high-dimensional data, leading to the creation of a branch of applied mathematics called Topological Data Analysis (TDA). Since then, TDA has been progressively adopted in biomedical research. Biological data collection can result in enormous datasets, comprising thousands of features and spanning diverse datatypes. This presents a barrier to initial data analysis as the fundamental structure of the dataset becomes hidden, obstructing the discovery of important features and patterns. TDA provides a solution to obtain the underlying shape of datasets over continuous resolutions, corresponding to key topological features independent of noise. TDA has the potential to support future developments in healthcare as biomedical datasets rise in complexity and dimensionality. Previous applications extend across the fields of neuroscience, oncology, immunology and medical image analysis. TDA has been used to reveal hidden subgroups of cancer patients, construct organizational maps of brain activity and classify abnormal patterns in medical images. The utility of TDA is broad and to understand where current achievements lie, we have evaluated the present state of TDA in cancer data analysis.

**Results:**

This article aims to provide an overview of TDA in Cancer Research. A brief introduction to the main concepts of TDA is provided to ensure that the article is accessible to readers who are not familiar with this field. Following this, a focussed literature review on the field is presented, discussing how TDA has been applied across heterogeneous datatypes for cancer research.

## 1 Introduction

The generation of large-volume, high-dimensional datasets is now routine across the biomedical sector. Healthcare data acquisition can range from whole-slide imaging containing billions of pixels (Yamamoto *et al.*, 2019) to single-cell transcriptomics on thousands of cells (Han *et al.*, 2020). By observing biological systems with increased details, we can improve our understanding of the mechanisms and forces driving disease. With this increase in dataset size comes a demand for big-data analytic tools capable of uncovering relevant information. Machine learning has proved a valuable solution within bioinformatics, excelling in predictive tasks on unseen data (Ching *et al.*, 2018; Kourou *et al.*, 2015). However, this branch of computer science can falter under more exploratory experiments designed to understand the nature of data. Underlying biological patterns can become difficult to observe following complex modelling (Alyass *et al.*, 2015). Unsupervised learning techniques, such as dimensionality reduction, can reduce all variables to a set of principal components, yet often loses important global or local structure in the process (Ayesha *et al.*, 2020; De Silva and Tenenbaum, 2003). Clustering analysis aims to identify groups of similar data points but can enforce artificial boundaries on biological phenomena (Cámara, 2017).

Topological Data Analysis (TDA) offers an alternate approach for big biomedical data analytics, obtaining robust representations of dataset structure according to the concept of topology (Carlsson, 2009). This branch of data science defines structure and shape within datasets by profiling the data as point clouds with a notion of distance or similarity in a co-ordinate free approach (Carlsson, 2014; Munch, 2017). Datasets sampled on the same biological systems using different technology platforms can therefore be compared directly (Lum *et al.*, 2013). To summarize the key structures underlying the shape of the data, TDA considers connections built between each data point over multiple scales, composing a continuous shape over the data. Structural features, which form the foundation of the data shape become apparent as they persist undisturbed over the different resolutions. This enables a framework that is insensitive to noise and to sampling metrics (Chazal and Michel, 2017). TDA is well suited to handle any high dimensional and messy data that may be present in biological analysis (Humphreys *et al.*, 2019; Liao *et al.*, 2019; Qaiser *et al.*, 2019; Saggar *et al.*, 2018).

The applications of TDA in biomedicine has extended across neurology (Lee *et al.*, 2012; Petri *et al.*, 2014; Saggar *et al.*, 2018; Sizemore *et al.*, 2018), gene-level and single-cell transcriptomics (Cho *et al.*, 2019; Lockwood and Krishnamoorthy, 2015; Nicolau *et al.*, 2011; Rabadán *et al.*, 2020; Wang *et al.*, 2019) , drug research (Benzekry *et al.*, 2015; Jiang *et al.*, 2016; Rietman *et al.*, 2017), evolution (Camara *et al.*, 2016; Chan *et al.*, 2013) and protein structural analysis (Cang and Wei, 2018; Kovacev-Nikolic *et al.*, 2016; Xia *et al.*, 2015). A broad overview discussing the interception of applied mathematics with biology using TDA is available in Amézquita *et al.* (2020), covering subcategories of biology including cell architecture, molecular biology, biological networks and evolution. Additional reading can be found in Camara *et al.* (2016) for a focussed discussion on TDA in the field of genomics, limited to gene-expression data. This current review article collates and summarizes literature implementing TDA for cancer research, addressing articles covering a heterogenous range of datatypes. Such applications can elucidate actionable discoveries hidden to more standard analysis, an example demonstrated in Rabadán *et al.* (2020) through integrative molecular analysis using TDA to uncover cancer-associated genes across different tumour types. Our review focuses on the applied aspect of TDA, considering the implementation of tools to the tasks of disease classification, prognostic prediction, treatment response profiling and patient stratification.

## 2 Background

TDA is an approach to data analysis, which uses techniques from topology, a field of mathematics concerned with the study of shape. There are three key properties of topology, which makes the application of TDA methods to the analysis of large datasets beneficial, these are discussed by Lum *et al.* (2013). Definitions of important terminology are provided in a glossary (Table 1).

1. Co-ordinate invariance Often datasets are represented in co-ordinate systems, which are not the definitively natural way to represent the data and certain methods may lead to different conclusions on the same datasets depending on the co-ordinate system chosen (Carlsson, 2009). Topological features are co-ordinate invariant, meaning that no matter which co-ordinate system is chosen to represent the data, the intrinsic topology of the shape of the dataset is identical. This property lends itself to the analysis of biomedical data, as it is often unclear as to which metrics are justified in a biological context. Variations in the methodology used to extract data within differing labs can lead to significant bias or offset between the datasets; the topological features of the datasets are, however, unaffected by bias or offset within the representations. Lum *et al.* (2013) demonstrate the co-ordinate invariance property by comparing microarray data across studies by using TDA methods to show common topological structure.
2. Deformation invariance Topological features of data are deformation invariant, which means that they do not change with respect to small deformations performed on the data (Lum *et al.*, 2013). A trivial example of this is that as humans, we are able to interpret the same meaning of the letters across differing font types. In the context of biomedical data, as our datasets grow larger there is an increasing probability that noisy experimental data will be present. While analysing the data with TDA techniques, we are better able to disregard the influence of noise. In biological systems, the stochastic nature of molecular processes can also introduce intrinsic noise, which can infer detrimental or beneficial consequences to cellular behaviour (Eling *et al.*, 2019). Multi-level topological representations found in TDA can provide summary statistics to discriminate dynamic molecular changes over time. (Cang *et al.*, 2020; Carlsson, 2017; Tran and Hasegawa, 2019).
3. Compressed representations Topological analysis of data produces a summary or compressed representation of a data shape. For example, a high-dimensional circle made up of infinite points, its topological representation will trade-off detail whilst maintaining the inherent topological properties by representing the circle as a finite number of points connected in a loop, e.g. an octagon (Lum *et al.*, 2013). In complex shapes, such as those that are existent in biomedical data, compressed representation can be used to help rapidly uncover hidden patterns and relationship in data (Amézquita *et al.*, 2020; Carlsson, 2017). Examples of complicated biological geometry analysed using TDA includes helical DNA structures (Meng *et al.*, 2020), brain imagery (Petri, 2014; Saggar *et al.*, 2018; Sizemore, 2018), 3D shapes in protein structure (Kovacev-Nikolic *et al.*, 2016) and branching behaviour of evolutionary trees (Camara *et al.*, 2016; Chan *et al.*, 2013; Humphreys *et al.*, 2019).

**Table 1.** A glossary of commonly used terminology in TDA

Simplicial complex	A simplicial complex is a set of simplices. Simplices generalize the concept of triangles according to their *k*-dimensions. A 0-simplex is a single point, a 1-simplex connects two points with an edge and a 2-simplex is a filled triangle.
Persistent homology	A method to compute the topological features of a space, shape or function. It works by considering the timeline over which data points will become connected across a changing distance parameter, known as the filtration.
Mapper	An algorithm that maps high-dimensional data to a low-dimensional network graph over the distribution of a lens function.
Filtration	Simplicial complexes are formed when edges are drawn between data points in space. The filtration is the sequence of simplicial complexes as they are born and die across the changing distance parameter during persistent homology.
Topological feature	Key structural information that summarizes the shape of data. These features persist throughout the filtration of simplicial complexes.
Birth and death	The point in a filtration when a topological feature first appears (birth) and where it ends (death).
Persistence barcode	A persistence barcode represents each topological feature as a horizontal line. The line begins at the birth time and ends at the death time.
Persistence diagram	A persistence diagram plots each topological feature obtained from filtration. The *x*-axis represents the birth time, and the *y*-axis represents the death time.

### 2.1 Persistent homology

**Fig. 1.**
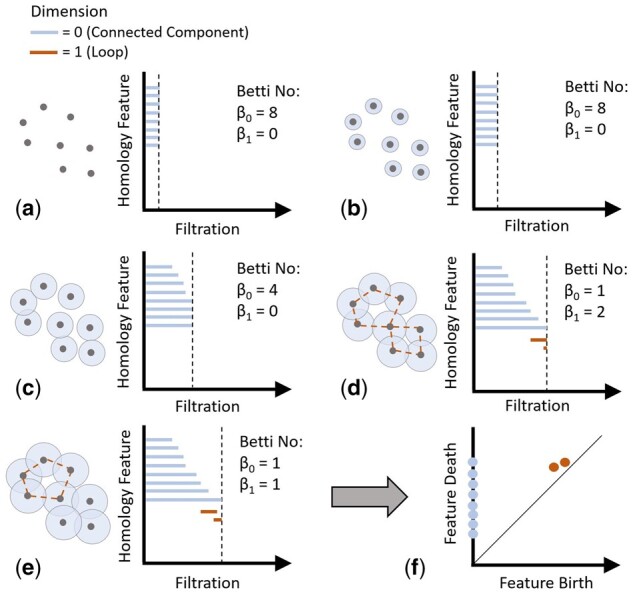
PH representation. (**a**) Point cloud data points shown alongside the persistence barcode representation. The vertical dashed line on the barcode indicates the filtration value at this point during the computation of PH. (**b**) Increase in the filtration value shown by increase in length of barcode bars and circles around data points. (**c**) Increasing filtration resulting in overlap between circles signifying connections between points (longer bars represent longer living features). (**d**) Formation of loops occur with filtration increase. Connections between points, which are within the loops are illustrated by dashed lines between the points. (**e**) One loop and connected component persists shown by the continuation of their barcode line. (**f**) An equivalent representation of the persistence barcode called a persistence diagram where the distance from the diagonal is the lifespan of the features. Adapted from Chazal and Michel (2017)

The first influential tool of TDA is referred to as persistent homology (PH) (Carlsson, 2009; Edelsbrunner *et al.*, 2000; Zomorodian and Carlsson, 2005). PH (Fig. 1) is used as a method to analyse the qualitative features of the topology of a dataset by encoding those features within an accessible diagrammatic representation. The topological features of data can be described as the number of connected components, loops and voids. By allowing for analysis of these features, even an abstract idea of the shape of data embedded in dimensions higher than three can be interpreted by practitioners wanting to gain actionable insights into the data space.

#### 2.1.1 Data in mathematical context

**Fig. 2.**
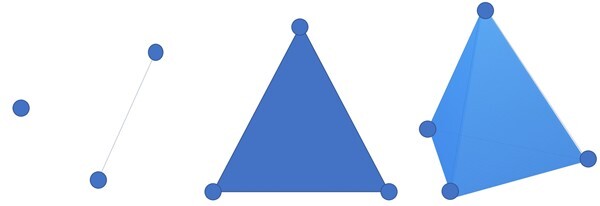
Simplices of increasing dimensions. From left, 0-simplex, 1-simplex, 2-simplex and 3-simplex

From a set of data points in a high-dimensional space, referred to as a point cloud (Fig. 1a), the shape cannot be interpreted by analysing those points independently of each other, i.e. no loops or voids can be found within the data space. Therefore, in its first step, PH represents the input data in a ‘connected’ way. The connectivity of points within a space is computed based on proximity of the point from one another. For a given distance matrix, if the distance between two points is no larger than a set threshold (also called a radius), which is a non-negative parameter, an edge is formed between them [Fig. 1(c–e)]. This allows us to represent our data as a collection of simplices (Fig. 2), which include vertices (0-simplex), edges (1-simplex), triangles (2-simplex), tetrahedron (3-simplex) and higher-dimensional polytopes. A collection of multiple simplices is referred to as simplicial complex.

#### 2.1.2 Persistence

The simplicial complex provides information about the topological features, such as connected components and loops. However, the homology of data may differ for different value of the distance threshold and it is not possible to determine which single threshold is the most representative of the topology of the data space. PH addresses this issue by considering several or all possible values of the parameter. As the value of the threshold increases, new simplices are included to the complex. Such a sequence of subcomplexes is referred to as ‘filtered simplicial complex’. PH records the value of the distance thresholds (filtration values) at which each feature is ‘born’ and ‘dies’, capturing how the homology of the complexes changes as the parameter values increases. This provides the lifespan of each feature indicating its persistence. Short-lived features are generally considered noise while long-lived features are considered robust features of the dataset (Carlsson, 2009). The upper-bound criteria for determining the final distance threshold is the maximal distance between any two samples in the dataset, the user can limit this to trade-off completeness for reduced computation time. Biological interpretation of the birth and death of features depend on the application, e.g. in 3D medical image analysis, such as MRI scans of brain artery trees, considering the loops, the number of birth/death values would indicate more looping within the brain artery trees, which itself has been used as a feature to help determine the age of the subject from MRI scans (Bendich *et al.*, 2016).

#### 2.1.3 Representation of persistent features

There are two methods of visually representing the PH, the persistence barcode and persistence diagram, both illustrated in Figure 1(e–f) (Chazal and Michel, 2017). The persistence diagram shows each topological feature as a point on a graph, the X- and Y-axis representing the filtration value at the birth and death of the feature, respectively (Fig. 1f). A persistence barcode represents each feature as a line of which the length represents the lifespan of the feature [Fig. 1(a–e)]. In both cases, the colour of the barcode or point indicates the type of topological feature. Here, in Figure 1, the zeroth dimension represents connected components and the first dimension representing loops. Figure 1(a–e) shows the effect of increasing the filtration value on the topological features, illustrated as circles of increasing radius around the point cloud samples. The number of features within the structure at any filtration value is referred to as the Betti value, where *β*_0_ is the number of structures within the 0D homology group (number of connected components) and *β*_1_ within the 1D homology group (number of loops). As individual points merge (any overlap between circles), the zeroth Betti number reduces until all points are connected as a homogeneous structure, where *β*_0_ = 1. As *β*_1_ represents loops, the formation of two loops (illustrated with dashed lines) in Figure 1d gives an increase of *β*_1_ to 2, one of which collapses in Figure 1e. If the filter value was to continue to increase, the final loop would also collapse, leaving a single connected component, thus, the barcode does not increase past the filter value at which the loop ‘dies’.

From the brain artery MRI scan example (Bendich *et al.*, 2016), PH allows us to extract features considering the shape and connectivity within brain artery trees at variable scales. Observing the presence of more loops, i.e. the *β*_1_ value, at a range of scales indicates to what extent the brain artery tree is connected via loops in the arteries, and the lifespan of those features indicates the loops’ size. By performing statistical analysis on the persistence diagram output, the mid-range filtration values were found to have the best correlation with the age of the subjects and little correlation was found with the most persistent features, demonstrating the importance of analysing homology at various scales.

By encoding the topological structure of data via PH both the persistence barcode and persistence diagram can provide insight into the understanding of the structure of data, allowing practitioners to form an idea of useful characteristics of the topology, e.g. long-lived connected components could indicate clusters of points within a dataset. Additionally, PH provides a new way to represent data when applied with data analysis techniques, such as machine-learning models. Vectorization techniques have been developed for persistence barcode and persistence diagram to standardize the representation of features from PH to allow for input to classification models (Pun *et al.*, 2018). Current vectorization techniques have resulted in varying degrees of success when used for classification tasks (Adams *et al.*, 2017). At present, it is not well-known which methods may be more discriminatory for particular applications; therefore, much research in the area considers new ways by which PH representations can be vectorized. One method of vectorization shown by Adams *et al.* (2017), maps the standard persistence representations to an image representation, opening up current state-of-the-art image classification machine-learning models to receiving PH representations as an input.

To make comparisons between persistence diagrams the bottleneck distance can be used, this is a value useful for describing the similarity between two persistence diagrams, i.e. each point on the diagram is matched with another on a separate diagram, or the diagonal if there is not an equal number of points, the smallest possible distance between the points is taken as the bottleneck distance (Fasy *et al.*, 2014).

### 2.2 Mapper

**Fig. 3.**
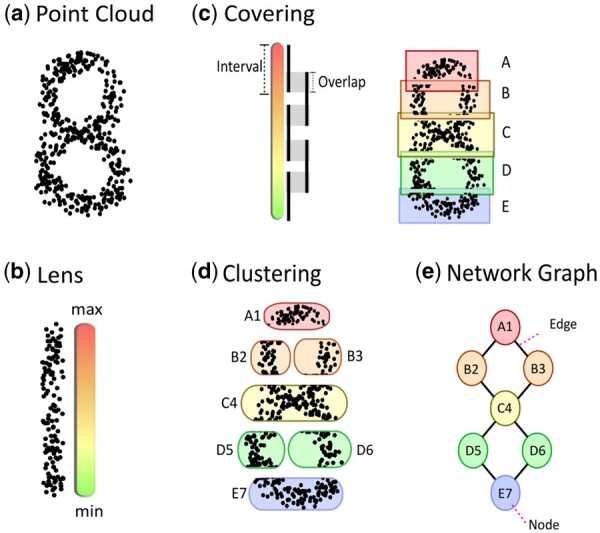
Details of the Mapper algorithm workflow. (**a**) A collection of data points (i.e. point cloud). (**b**) The point cloud is represented by a vector of real numbers obtained via the lens function. (**c**) A cover is built on the lens by dividing the function across a set of overlapping intervals. The point cloud is binned into the intervals according to the lens value of each data point. (**d**) Clustering is performed within each interval on the original space. (**e**) A network graph is built. Each node corresponds to a cluster of the point cloud. Edges join nodes together if the clusters overlap

The second implementation of TDA used in biomedical data analysis is referred to as Mapper (Singh *et al.*, 2007). The Mapper algorithm can be used to build graph-based landscapes of high-dimensional data capturing topological and geometric information. The sequence of steps involved in the Mapper algorithm is illustrated in Figure 3. Similarly as with PH, the input is a dataset considered as a point cloud, where each observation represents a measurement set in space. Mapper is applied to interpret the collective shape of the point cloud (Fig. 3a).

In the first step, the data points are grouped together according to their geometric proximity and according to the values of the lens function, also called the filter function (Fig. 3b). The lens consists of a vector of real numbers corresponding to each data point in the point cloud. The choice of the lens function allows the user to consider the structure of the dataset over a broad perspective or a more focussed view considering specific research questions. The lens function can take the form of projections used in statistics and machine learning to observe the core components of the dataset (e.g. Mean, Principal Component Analysis and L-infinity centrality), it may compute some geometric properties (e.g. density or centrality) or may be defined by a user as a property they want to study (e.g. patient survival). The introduction of lens functions based on biologically informative aspects of the data can provide further insight on the system being investigated. Examples have included survival outcomes of breast cancer patients (Lum *et al.*, 2013), or the deviation of samples from a healthy state model (Kyeong *et al.*, 2015; Nicolau *et al.*, 2011). Based on the lens values, the data are partitioned into overlapping intervals (bins) to form a cover (Fig. 3c). The number of intervals and percentage of overlap are parameters, which dictate how the cover is divided. The cover can be understood as the resolution over which the structure of the data is observed. The concept of topological persistence is present in the cover of Mapper, as geometric features that are intrinsic to the structure of the dataset will be present over multiple resolutions. Lum *et al.* (2013) recommend experimenting with the settings of Mapper to identify interesting shapes in the network structure. Following the covering, the data within each bin are clustered internally (Fig. 3d). Common clustering algorithms used include Hierarchical Agglomerative clustering and DBSCAN.

The clusters obtained across all the bins are then converted into a graph, where each node represents a single cluster, and all overlapping clusters containing shared data points are connected by an edge (Fig. 3e). Multiple parameters must be considered in the implementation of Mapper, as the final geometric output is sensitive to the choice of distance metric, lens, clustering algorithm, number of intervals and percentage of overlap between intervals (Carriere *et al.*, 2018). The final network graph can be coloured by the lens function or by any important features present in the dataset.

## 3 TDA for cancer research

TDA excels at identifying patterns within large and complex volumes of data that are collected for cancer research. To underline the value of TDA in biomedical data analysis, we present a focussed review of TDA techniques for cancer research. This will summarize how PH and Mapper have been applied to support advancements in oncology. A literature search across the Scopus database, Google Scholar and using manual cross-referencing identified 80 high-quality papers applying TDA to the field of Biomedicine. As the scope of this review investigates applications of TDA to cancer research, we restricted the literature review collection to 31 papers implementing either PH or Mapper to perform data analysis tasks in oncology. The search was performed over October 1, 2019 to June 1, 2020. Key search terms include (‘TDA’ OR ‘topological data analysis’ OR ‘persistent homology’ OR ‘Mapper’) AND (‘biomedicine’ OR ‘health’ OR ‘biology’ OR ‘cancer’ OR ‘oncology’).

### 3.1 Persistent homology

#### 3.1.1 Disease classification

PH’s ability to encode the topological structure of data has led to research into ways in which the technique can be employed within cancer research to extract topological-based features from medical image data. Performing classification tasks on whole-slide images, Singh *et al.* (2014) and Qaiser *et al.* (2016) investigated how PH-based features can be used for tumour cell classification tasks. Their findings suggest that combining features extracted via PH and features based on image texture [features considering pixel intensity and the spatial relationships of pixels within the images (Kather *et al.*, 2016)], offer improved performance over using one type of feature for tumour cell classification. That indicates the PH-based features can add supplementary information on cell morphology. To classify the tumours within breast cancer whole-slide images, Singh *et al.* (2014) extracted topological summaries using the distance between the cell nuclei’s positions as the filtration value for PH, then performed classification using distance weighted discrimination (DWD). The proposed features obtained similar classification performance to appearance-based features extracted using sparse dictionary learning (Mairal *et al.*, 2010), a technique used to group subsections of the image by appearance similarity, then taking the number of subsections within each grouping to be a feature. Furthermore, in some cases, when used to discriminate between the breast cancer subtypes with DWD, the performance was improved by combining the PH-based features and the appearance-based features. For example, in one task, when distinguishing between the Luminal B and HER2 subtypes, combining the features added 8% to the classification accuracy. Qaiser *et al.* (2016) built a PH profile using a filtration value based on greyscale pixel intensity to segment colorectal tissue whole-slide images into areas of tumorous and healthy areas. When compared against a Convolutional Neural Network (CNN) model, which is currently considered state-of-the-art for the task of image classification (Anwar *et al.*, 2018; Dabeer *et al.*, 2019; Iqbal *et al.*, 2018), features extracted from the PH profiles used as input to a *K*-nearest neighbours model resulted in the best classification performance across datasets with an *F*1-score of 0.7804 compared to 0.7071 when using a CNN model for classification.

Using features derived from the PH bottleneck distance, Adcock *et al.* (2014) present a framework of how topological features can be used to classify image data applied to the task of classifying hepatic liver lesions from CT scans. The bottleneck distances from each PH barcode to the others were considered features in a support vector machine classifier, resulting in a classification accuracy of 80.56% over the entire dataset. However, other studies referenced (Kurtz *et al.*, 2014) were able to achieve up to 95% accuracy with standard features, e.g. semantic annotation and texture-based features. Thus, performance may benefit from experimentation using topological features in combination with standard features, which was not explored in this study.

#### 3.1.2 Prognostic prediction

A better understanding of the relationship between tumour appearance on medical image data and genetic profile of the tumour has the potential to improve patient care within larger populations, with advances in radiogenomics leading to quicker, less-invasive prognosis using routine medical imaging techniques rather than relying on microarray data for accurate prognoses (Yeh *et al.*, 2019). In two studies (Crawford *et al.*, 2019; Kim *et al.*, 2019), it has been investigated how patient prognoses could be predicted using features extracted from images using PH. In Crawford *et al.* (2019), a novel statistical feature, which uses PH named the smooth Euler Characteristic Transform (SECT) was applied to quantify the structure of glioblastoma tumours within MRI images. Rather than encoding PH as a persistence diagram, SECT represents PH as a single integer by alternating the sum of the number of connected components/holes to *n*-dimensions, allowing for application to regression analysis as performed within the study to predict two types of outcomes for glioblastoma, the disease-free survival and overall survival. The authors found the PH-based feature to be the best predictor for disease-free survival, performing optimally over gene-expression data and the standard volumetric and morphometric features.

In contrast to the regression analysis, Kim *et al.* (2019) investigated how well features found using PH could discriminate between MRI scans of those patients with and without a rare genetic deletion event associated with a better glioma prognosis/survival rate (i.e. classifying an MRI image as someone who had or had not undergone 1p/19q codeletion to act as a surrogate for determining prognosis). The topological features used were values extracted from PH barcodes computed over the MRI scans, using pixel intensity as a filtration value, e.g. the values at which features are formed, bar lengths and distributions of the deaths in relation to the filter value. The authors compared the classification performance of both random forest and linear regression classifiers when applying TDA-based features, features extracted from the image representation learned by a CNN model and numerous texture-based features. One such texture-based feature, the Gray-Level Co-Occurrence Matrix, calculates how often pixels of a specific value occurs relative to a paired pixel with a common position and intensity (Kather *et al.*, 2016). The performance, measured as area under the receiver operator curve (AUROC), of the TDA-based features outperformed the texture-based features and performed comparably to the CNN, with the best AUROC performances of 0.710, 0.660 and 0.708, respectively. However, when the feature selection method, k-Top Scoring Pairs, was used to keep only the most discriminative features, those from the CNN outperformed the TDA-based features giving an AUROC of 0.718.

#### 3.1.3 Treatment response

When developing targeted therapy cancer treatments, transcription factor proteins that can contribute to the alteration of gene-expression patterns leading to tumour growth are identified to allow for focussed development of drugs that target those proteins likely to be successful in targeted therapy (Sammak and Zinzalla, 2015). Furthermore, as the transcription factors often do not act independently (Amoutzias *et al.*, 2008), protein–protein interaction (PPI) networks are used to represent the complex relationships between proteins and prove useful when identifying disease pathways. PH to compute the topological characteristics of PPI networks has shown potential when used to identify target proteins (Benzekry *et al.*, 2015; Rietman *et al.*, 2017). Similar to the ‘degree-entropy’ metric introduced in Breitkreutz *et al.* (2012), which was correlated to the 5-year survival of prostate cancer patients, Benzekry *et al.* (2015) proposed that the Betti numbers from PH could be used as a measure of cancer PPI network complexity by quantifying the number of cycles/loops present in the network. The PH is computed using the shortest path between two nodes as the filtration value (i.e. two proteins are considered connected in the PH representation if there is a minimum number of edges between the nodes less than or equal to the filter value). In biological terms, the protein nodes within the loops are seen to be indicative of disease pathways. A linear correlation was shown between the PH in the protein–protein network data and 5-year survival of patients across multiple cancers with higher sensitivity than the degree-entropy complexity measure from Breitkreutz *et al.* (2012). Potentially important proteins in targeted therapy can be identified by removing proteins from the network and measuring the most significant reduction in the number of loops. Following on from this work, Rietman *et al.* (2017), suggests that the best therapeutic targets for low-grade glioma vary between patients and that this type of analysis using PH with protein–protein networks in a precision medicine setting, could help improve molecular therapy drug selection tailored to an individual by predicting the best treatment response.

Other than applications to treatment response analysis with PPI networks, PH was used by DeWoskin *et al.* (2010) as a technique to help characterize comparative genomic hybridization (CGH) profiles making use of entire chromosome segments of the profile, viewing the CGH data as a collective, contrary to other CGH analysis methods that identify segments or specific clones associated with a disease (Picard *et al.*, 2005). The CGH profiles used were from breast cancer patients who had undergone different treatments with the aim to observe if there was a significant difference between the profiles of patients for which recurrence occurred and those it did not. The method used a sliding window technique to map the CGH data to a point cloud lying in a dimension equal to the window’s length. For each patient, the number of connected components was calculated, and statistical analysis showed that the profiles of chromosomes 8 and 11 were significantly different for those recurrent and non-recurrent patients who did not undergo chemotherapy. However, no significant difference was found for patients who did undergo chemotherapy.

### 3.2 Mapper

#### 3.2.1 Patient stratification

Patient stratification is defined as the separation of a population of patients with the same disease into subgroups of individuals that share common molecular or clinical features (Jameson and Longo, 2015). It is a procedure often employed in cancer research to benefit healthcare outcomes by tailoring treatment to specific cohorts of patients, consequently improving disease outcomes and reducing the use of ineffective drugs (Trusheim *et al.*, 2007). The standard bioinformatic techniques used to differentiate patient cohorts are clustering algorithms, such as hierarchical clustering or *K*-means (Parimbelli *et al.*, 2018). While popular, these methods suffer from poor performance when applied to data with high levels of noise or non-linear shapes, which is common in biological data (de Souto *et al.*, 2008; Rodriguez *et al.*, 2019). Importantly, these techniques can force artificial membership of patients into discrete disconnected clusters (N’Cir *et al.*, 2015). This has led to the implementation of Mapper to produce fine-detail maps of patient data distribution, accounting for most Mapper applications in healthcare (Lee *et al.*, 2017; Li *et al.*, 2015; Nicolau *et al.*, 2011; Nielson *et al.*, 2015; Rabadán *et al.*, 2020). By employing partial clustering in Mapper, phenotypes can be represented over a higher resolution as relationships between patients across clusters are conserved. Individuals can be present in multiple clusters simultaneously, reflecting shared traits across phenotypes. Mapper can also reflect aspects of geometry or biology beyond the breakup into clusters, improving the interpretability of patient stratification.

Mapper has been found to improve relevant stratification of cancer from standard clustering analysis, identifying subgroups hidden to classical hierarchical clustering according to gene-expression data (Mathews *et al.*, 2019b). In breast cancer, papers by Lum *et al.* (2013), Mathews *et al.* (2019a) and Nicolau *et al.* (2011) have identified focussed subgroups according to specific research questions by integrating disease-relevant information with the Mapper algorithm. In Lum *et al.* (2013), the lens function incorporated survival data to identify a subgroup of patients that survived and had low expression of the oestrogen-receptor gene. In Nicolau *et al.* (2011), patients were stratified according to a calculation based on their deviation from healthy tissue. The study demonstrated how Mapper highlighted which patients had increased gene expression corresponding to a diseased state, and subsequently identified a novel subgroup of oestrogen-receptor positive patients with 100% overall survival with no recurrence and no death from disease. A paper by Mathews *et al.* (2019a) based the lens function on differentiation scores of mammary cell types. They identified seven clusters of breast cancer patients in the Mapper graph according to disease functionality. Finally, Mathews *et al.* (2019b) presented an additional method to incorporate biological relevance in Mapper, by restricting the point cloud to genes specific to the TP53 signalling pathway and calculating a network-based distance metric between samples based on pathway weights. This produced a network of TCGA sarcoma patients defined by different states of active and inactive members of the TP53 signalling network.

## 4 Summary

### 4.1 TDA tools available

PH and Mapper can be implemented through publicly available tools, some examples include Gudhi, a C++ library with Python bindings for PH computation (Project, 2020) (https://gudhi.inria.fr/) and KeplerMapper (Van Veen *et al.*, 2019), which provides an implementation of the Mapper algorithm in Python (https://kepler-mapper.scikit-tda.org/index.html). Both TDA techniques are found in ‘giotto-tda’ (Tauzin *et al.*, 2021), a Python library that implements PH and Mapper in a machine-learning compatible framework (https://giotto-ai.github.io/gtda-docs/0.3.0/index.html).

Important research is still required on the limitations of both the Mapper algorithm and PH tools, touching on aspects of parameter optimization, feature extraction and statistical analysis. This includes reducing the number of decisions required for parameter selection and the introduction of robust quality measures for Mapper graphs or confidence regions for topological features. Addressing these issues will improve the accessibility of TDA methods for non-experts, such as healthcare practitioners. Currently, there is no comprehensive assessment of TDA performance compared to existing unsupervised machine-learning techniques. Understanding where the weaknesses of current methods lie is a key to combine the strengths of both machine learning and TDA methodology for enhanced data analysis. A solid mathematical foundation has been built for TDA, and progress in the development of specialized tools for biological problems can prove a key to improved integration of TDA in the bioinformatics workflow.

### 4.2 Limitations of TDA within cancer research

With increasing dimensionality, the important features driving data structures can become lost amongst noise and outliers. TDA currently stands as a new approach by which to tackle high-dimensional biomedical data analysis. It is not restricted by data type or technological platform due to its co-ordinate free analysis and is therefore consistent across applications and datasets. Although promise has already been shown by applications of TDA within cancer research, the field is still in its infancy and will benefit from robust analysis of the topological features when validating links with biological functionality. For example, the biological interpretation of structural features from PH depends on the dataset on which it is applied. However, more research needs to be carried out before conclusions can be made about the biological significance of those encoded features beyond speculation. Currently, the research is limited to a subset of cancer types and applications to a broader range will help solidify TDA as a tool for exploring data structure and consensus on how the TDA tools are best applied. For example, with continued research across datasets with the Mapper algorithm, choices of hyperparameters can be identified which are preferential when applied to specific types of analysis, given the limitation that it is difficult to determine which hyperparameters may reveal important structure.

### 4.3 Future development of TDA within cancer research

Future development of TDA methods for drug discovery in cancer treatment is promising. In addition to preliminary studies from Benzekry *et al.* (2015), which applied PH to PPI networks determining potential molecular therapy targets, Mapper has been incorporated into the pipeline of *in silico* drug discovery by supplementing the analysis of high-throughput screening. Standard analyses typically employ unsupervised hierarchical clustering to separate compounds with similar chemistry into compound families (Vogt and Bajorath, 2012), but restricted to compound families and can lose local information on individual data points. Jiang *et al.* (2016) proposed using Mapper to avoid this by creating a compound network corresponding nodes to compound clusters and edges between nodes displaying similar inhibition profiles of compounds. This allowed for the identification of compounds toxic to multiple myeloma cell lines demonstrating the utility of this method to support targeted treatment in precision medicine, which may prove invaluable for the drug development process with further research to back it as this is currently the only article to explore the technique.

Incorporating TDA within longitudinal studies exploring how the topology of oncological datasets evolves with time would be of interest for future research for insights into cancer progression. For example, a longitudinal study that followed the progression of malaria infection (Torres *et al.*, 2016) proved to illustrate how variation in the position of samples within the Mapper graph could indicate resilience or susceptibility poor outcomes of the diseases, applying the same methodology to cancer progression could give similar insights by mapping a ‘disease space’.

A direct application of Mapper to scRNA-Seq analysis with important biological findings has been reported by Lee *et al.* (2017), improving genomic profiling of glioblastoma tumours. Mapper was used to identify concurrent expression features within individuals with multiple tumours. However, applications to cancer research are limited, with only Wang *et al.* (2019) demonstrating how gene co-expression modules in melanoma can be represented within the Mapper network graph to provide visualization of cell diversity while maintaining the concept of relationships between nodes of the Mapper graph. Exploring scRNA-Seq analysis further with TDA allows practitioners to explore data from other perspectives that the standard dimensionality techniques do not allow.

## Funding

This work was supported by the Department for the Economy (DfE), Northern Ireland, UK.

*Conflict of Interest*: none declared.
